# Application of a New Carbohydrate Food Quality Scoring System: An Expert Panel Report

**DOI:** 10.3390/nu15051288

**Published:** 2023-03-05

**Authors:** Kevin B. Comerford, Adam Drewnowski, Yanni Papanikolaou, Julie Miller Jones, Joanne Slavin, Siddhartha S. Angadi, Judith Rodriguez

**Affiliations:** 1 OMNI Nutrition Science, Davis, CA 95616, USA; 2 Center for Public Health Nutrition, University of Washington, Seattle, WA 98195, USA; 3 Nutritional Strategies Inc., Nutrition Research & Regulatory Affairs, Paris, ON N3L OA3, Canada; 4 Department of Nutrition and Exercise Science, St. Catherine University, St. Paul, MN 55105, USA; 5 Department of Food Science and Nutrition, University of Minnesota, St. Paul, MN 55105, USA; 6 Department of Kinesiology, University of Virginia, Charlottesville, VA 22903, USA; 7 Department of Nutrition & Dietetics, University of North Florida, Jacksonville, FL 32224, USA

**Keywords:** carbohydrate quality, fiber, free sugars, whole grain, sodium, potassium, dietary guidelines for Americans, Thrifty Food Plan, culturally inclusive dietary patterns

## Abstract

Carbohydrate foods (≥40% energy from carbohydrates) are the main source of energy in the US diet. In contrast to national-level dietary guidance, many regularly consumed carbohydrate foods are low in fiber and whole grains but high in added sugar, sodium, and/or saturated fat. Given the important contribution of higher-quality carbohydrate foods to affordable healthy diets, new metrics are needed to convey the concept of carbohydrate quality to policymakers, food industry stakeholders, health professionals, and consumers. The recently developed Carbohydrate Food Quality Scoring System is well aligned with multiple key healthy messages on nutrients of public health concern from the 2020–2025 Dietary Guidelines for Americans. Two models are described in a previously published paper: one for all non-grain carbohydrate-rich foods (e.g., fruits, vegetables, legumes) known as the Carbohydrate Food Quality Score-4 (CFQS-4), and one for grain foods only known as the Carbohydrate Food Quality Score-5 (CFQS-5). These CFQS models provide a new tool that can guide policy, programs, and people towards improved carbohydrate food choices. Specifically, the CFQS models represent a way to unify and reconcile diverse ways to describe different types of carbohydrate-rich foods (e.g., refined vs. whole, starchy vs. non-starchy, dark green vs. red/orange) and make for more useful and informative messaging that better aligns with a food’s nutritional and/or health contributions. The present paper’s aims are to show that the CFQS models can inform future dietary guidelines and help support carbohydrate food recommendations with other health messages aimed at promoting foods that are nutrient-dense, fiber-rich, and low in added sugar.

## 1. Introduction

The 2020–2025 Dietary Guidelines (DGA) noted that Americans have low intakes of whole grains, fiber, potassium, vegetables, and fruits, along with excess intake of refined grains, sodium, added sugars, and saturated fat [1]. These dietary patterns have been linked to higher risks for obesity, diet-related chronic diseases, and several other public health concerns across all life stages. Almost three-fourths of the US population are overweight or obese, and an even higher percentage suffers from one or more nutrient inadequacies [1]. There is a clear need to educate, encourage, and empower Americans toward making higher-quality food choices that provide optimal nutritional value. Carbohydrate foods (CFs) are a useful starting point since they contribute to roughly half of the energy in US dietary patterns. However, the quality of CFs in US dietary patterns is highly variable, and most Americans are regularly overconsuming CFs that are low in fiber and/or high in added sugars and sodium [2]. New metrics of carbohydrate quality would aid policymakers, industry stakeholders, health professionals, and consumers to better understand the importance of CF quality and the role that higher-quality CFs can play in healthy, affordable, and diverse dietary patterns.

The 1990 National Nutrition Monitoring and Related Research Act requires that the United States Department of Agriculture (USDA) and Department of Human and Health Services (DHHS) publish DGAs at least every 5 years with the intention of promoting the nutrition and health of the general public [3]. The most recent DGA, published in 2020, provides over 150 pages of information on nutrient intakes, foods and beverages, and dietary patterns that promote health and prevent chronic disease, yet there is little mention of carbohydrate quality aside from the recommendations to “avoid added sugars” and “increase dietary fiber”, along with a few specific food group recommendations such as to favor “dark green; red and orange” vegetables and “whole fruit”, and “make half your grains whole grains” [1]. Considering that CFs contribute a significant amount of energy to US dietary patterns [2], additional guidance tools for selecting CFs could help consumers to improve their diet quality and overall health.

While the DGA does provide a Recommended Daily Allowance (RDA) for carbohydrates of 130 g and an acceptable macronutrient distribution range (AMDR) of 45–65% of total calories from carbohydrates [1], future dietary guidelines would be well-served by utilizing carbohydrate quality metrics that use both quantitative and qualitative measures to inform their guidance. A major problem with putting this recommendation into action has been a lack of consensus on how to identify high-quality CFs, as there is much consumer confusion regarding the harms and benefits of CFs, with some mainstream beliefs trending towards consuming diets made up almost entirely of CFs (e.g., fruitarian diet), and others based on consuming very little (e.g., ketogenic diet) or removing one or more sources (e.g., grain-free diets) of carbohydrates from the diet. None of these types of dietary patterns are compatible with the recommendations of the current DGA, primarily because CFs from each carbohydrate-containing food group contribute several essential micronutrients that are needed to meet the nutrient demands of nearly all populations [4]. For this reason, the 2020–2025 DGA recognizes the nutritional value of refined grains in healthy dietary patterns, since refined grains contribute roughly 40% of dietary fiber in US diets [5], and these foods are also often fortified or enriched with various vitamins and minerals that help large portions of the population to meet their micronutrient needs [6,7]. Therefore, the DGA recommendation to make “half your grains whole grains” suggests that consuming up to 50% of grains from non-whole grain sources (e.g., fortified or enriched refined grains) may also play a critical role in helping Americans to achieve adequate nutrient intake.

To address the unique and diverse roles that CFs can play in healthy, affordable, and culturally inclusive dietary patterns, the present manuscript was developed by members of the Quality Carbohydrate Coalition—Scientific Advisory Council (QCC-SAC), which is a group with expertise in carbohydrate research, epidemiology, nutrient profiling, and cultural competency. This report builds on previous QCC-SAC work that identified several guiding principles for the development of CF quality metrics [8,9]. The goal of these principles was to develop a scoring system that included multiple components related to CF quality, such as fiber, free sugars, sodium, and potassium (all of which are recognized as nutrients of public health concern), and also in the case of grain foods, a component assessing whole grain content—which is a recommended dietary component in the 2020–2025 DGA. This earlier work recognized that a range of CFs come from all plant-based food groups (grains, vegetables, fruits, and legumes) and that their qualities (e.g., energy content, nutrient content, bioactive food components, and impacts on health) may differ considerably both within and among food groups. Therefore, a more nuanced approach to assessing CF quality than what currently exists is necessary to improve dietary guidance. Other findings from this earlier work emphasized that new metrics for assessing CF quality should aim to align with dietary guidelines, be adaptable to new evidence, be culturally inclusive, and be easy to use [8,9].

The present work aims to demonstrate how the Carbohydrate Food Quality Scoring System, which has produced two models to date, the Carbohydrate Food Quality Score-4 (CFQS-4) for non-grain CFs and the Carbohydrate Food Quality Score-5 (CFQS-5) for grain-based CFs, can inform future dietary guidelines and help align CF recommendations with other health-promoting messages such as aiming for more nutrient-dense, fiber-rich, and low sugar foods. These new evidence-based tools can also provide an opportunity to examine how CFs, within and among different food groups, align with existing DGA recommended dietary patterns, as well as with a variety of more culturally diverse dietary patterns that incorporate staple CFs (e.g., cassava, calabaza, taro, amaranth, plantains). Ultimately, the application of the CFQS models to different dietary patterns (e.g., DGA-style healthy dietary patterns, USDA Thrifty Food Plan, and culturally inclusive dietary patterns) can help guide healthier CF intake across a spectrum of different options ranging from fruits, vegetables, and legumes, to whole, enriched, or fortified grains, since these foods can all contribute multiple nutrients that are chronically underconsumed by Americans (e.g., fiber and potassium) while simultaneously being low in those that are consistently overconsumed (e.g., free sugar and sodium).

## 2. Materials and Methods

### 2.1. Carbohydrate Food Quality Scoring System

The development of the Carbohydrate Food Quality Scoring System is described in an earlier publication by the QCC-SAC [9]. In brief, two metrics—the CFQS-4 and CFQS-5 were developed to assess CF quality. This scoring system recognizes the value by using a four-point scale (i.e., CFQS-4) based on four nutrients of concern to assess the quality of non-grain CFs and a five-point scale (i.e., CFQS-5) based on the same nutrients of concern along with whole grain content for assessing the quality of grains foods. For non-grain CFs, the maximum score is four points (CFQS-4: with points based on meeting threshold measures of fiber, free sugars, sodium, and potassium); for the grain group, the maximum score is five points (CFQS-5: with points based on meeting threshold measures of fiber, free sugars, sodium, potassium, and whole grains) [9].

### 2.2. DGA/USDA and Culturally Inclusive Carbohydrate Food Tables

The 2020–2025 DGA outlines three dietary patterns that serve as frameworks to help individuals to follow a healthy dietary pattern and meet key nutrition recommendations. These patterns include Healthy US-Style, Healthy Vegetarian, and Healthy Mediterranean-Style [1]. The USDA’s Thrifty Food Plan represents affordable food and beverage options that support the Healthy US dietary patterns and is based on the cost to purchase groceries for a family of four while meeting the nutritional requirements of a person consuming a healthy, budget-conscious diet at home [10]. The Thrifty Food Plan informs the Supplemental Nutrition Assistance Program (SNAP) benefits for a household of four people and is used to determine the maximum benefit amounts for households of varying sizes.

Approximately 20 foods were selected to represent commonly consumed CFs in the United States, and these were condensed into a single table representing all four US-centric dietary patterns (Healthy US-Style, Healthy Vegetarian, Healthy Mediterranean-Style, and Thrifty Food Plan), since all these dietary patterns share similar CF recommendations (e.g., aim for 45–65% kcal from carbohydrates and a similar number of daily servings of fruits, vegetables, and grains).

Four additional tables were created to reflect CFs commonly found in culturally relevant eating patterns. According to US Census data, African American, Latino/Latin American, Asian American, and Native American are among the largest recorded ethnic groups [11]; thus, each table reflects CFs commonly consumed in alignment with these cultural eating patterns. Although each table reflects approximately 20 popular CFs commonly consumed by given racial/ethnic groups, these foods are not necessarily reflective of every cultural/ethnic group within each specified region; rather, the tables serve as examples of how culturally inclusive food patterns can be overlayed with DGA recommended dietary patterns and the CFQS models. Following inclusion criteria previously published by Drewnowski and colleagues [9] to develop the CFQS-4/CFQS-5 models, only foods with ≥40% energy from carbohydrates per 100 g dry weight were included in the culturally inclusive food tables. Foods containing < 40% carbohydrate per 100 g dry weight were excluded. CFs for each table were validated by a combination of Common Foods & Flavors lists from Oldways [12,13,14], a nonprofit that provides resources and inspiration on heritage-based diets, as well as Member Interest Groups of the Academy of Nutrition and Dietetics representing these populations (National Organization of Blacks in Dietetics and Nutrition (NOBIDAN), Latinos and Hispanics in Dietetics and Nutrition (LAHIDAN), as well as Women and Girls [of African Descent] Advancing Nutrition Dietetics and Agriculture (WANDA).

### 2.3. DGA/USDA Aligned Menu Modeling

Using the CFs listed in the DGA/USDA food table, three one-day menu models were developed to align with the three different DGA healthy dietary patterns (Healthy US-Style, Healthy Vegetarian, and Healthy Mediterranean-Style) to demonstrate the utility of the CFQS models when applied to dietary patterns that meet current national dietary guidelines. A fourth menu model and associated cost analysis were developed in alignment with the USDA Thrifty Food Plan. As part of the DGA menu modeling, tables are provided that align gender, age, and activity level to calorie and nutrient needs; thus, each menu was based on requirements for a woman, aged 20–50 years, consuming 2000 kcals/day. Nutrients of interest included three macronutrients, carbohydrates, protein, and fat; fiber; saturated fat; added sugars; calcium, potassium, and sodium; vitamin D. To reduce the risk of chronic disease, the DGAs have established upper limits for saturated fat, added sugars, and sodium in a healthy dietary pattern, as overconsumption of these nutrients makes it difficult for individuals to meet food group and nutrient recommendations while staying within calorie needs [1]. Furthermore, dietary fiber, calcium, potassium, and vitamin D are under-consumed across the US population, and low intakes are associated with detrimental health outcomes and increased risk of chronic disease [1]. For this reason, these nutrients have been identified as dietary components of public health concern and have been included in the present menu models.

The DGA establishes dietary patterns at 200-kilocalorie (kcal) intervals (i.e., 1800, 2000, 2200, and 2400); therefore, a deviation of +/− 100 kcals was considered acceptable (i.e., 1900–2100 kcals) to most closely support a 2000-kcal pattern and avoid encroaching upon lower or higher DGA calorie intervals.

Following the 2020 Dietary Guidelines Advisory Committee (DGAC) food pattern modeling protocol for ages 2 years and older, all nutrients of interest achieved 90% or greater of the Recommended Daily Allowance (RDA) or Adequate Intake (AI), with the exception of vitamin D [15]. Typically, population-based vitamin D intakes are well below the established nutritional recommendations (i.e., RDA) for a respective pattern [16,17]. On average, 46% of the RDA for vitamin D is met with the Healthy US-Style Dietary Pattern and 38% is met with the Healthy Vegetarian Dietary Pattern. Criteria were established to meet or exceed the population-based intake averages for vitamin D in each menu.

Each DGA dietary pattern (i.e., Healthy US-Style, Healthy Vegetarian, and Healthy Mediterranean-Style) and the USDA Thrifty food plan provide recommended daily and weekly intake amounts, in ounce or cup-equivalents, of five major food groups including vegetables, fruits, grains, dairy, and protein foods [1,10]. Given those recommendations for several sub-food groups (e.g., red and orange vegetables, eggs) are provided in amounts per week, yet the models reflect a one-day diet, nutrition criteria were established for the five major food groups in amount per day. Criteria were established to meet 90% of the recommended daily serving for each food group.

### 2.4. Modeling Software and Menu Creation

All menu models were created using ESHA Food Processor 11.11.32, version 11.11.0. [18]. Each one-day menu follows precedence from USDA menu modeling and thus includes three meals and two snacks (i.e., breakfast, morning snack, lunch, dinner, and evening snack) and adheres to the described kcal and nutrient requirements for the target population. All menus were created with foods across the food supply that was accessible and appropriate for the specific diet in question (i.e., Healthy US-Style, Healthy Vegetarian, or Healthy Mediterranean-Style). Furthermore, the selected food items were chosen to meet nutrient needs without exceeding total calories or overconsumed nutrients. Selected food items were input into the menu modeling software, and food items and serving sizes were further iterated based on the software’s nutritional output. Iterations were made until nutrient needs were satisfied. For all menus, CFQS scores were added after the software generation using CFQS scoring criteria, with the energy and nutrient content of foods from the Food and Nutrient Database for Dietary Studies (FNDDS) and Food Patterns Equivalents Database (FPED).

CFs with a CFQS of ≥3 were prioritized in each menu, while scores of 1 and 2 were also included due to their contributions to meeting nutrient requirements as well as their contributions to healthy, diverse, affordable, and culturally appropriate eating patterns. While important dietary factors such as enrichment and fortification are not captured in current CFQS models, the inclusion of enriched and fortified grains in the menu plans, as well as culturally relevant CFs that may have a lower CFQS, allow for foods with lower scores to still provide meaningful contributions to one or more components of healthy eating patterns. Thus, a wide variety of CFs across the food supply and of varying nutritional value were strategically featured in each menu to align with the caloric and nutrient parameters of each dietary pattern.

### 2.5. Thrifty Food Plan Cost Analysis

The Thrifty Food Plan, 2021 Market Baskets, represents foods and beverages and associated costs that support the Healthy US dietary pattern [10]. Given price variability among food ingredients as a result of individuals’ purchasing behavior (e.g., location of purchase, purchasing items in season, in bulk, or on sale), the cost analysis was conducted using the 2021 Market Baskets which reflect the cost of the Thrifty Food plan at June 2021 prices. The Thrifty Food Plan provides weekly and monthly costs intended to support budget-conscious groceries for a four-person household. For this research, an adjustment of 20 percent was applied to reflect a one-person household as suggested by the USDA [19]. Costs per food item were calculated using the quantity (pounds) and cost (dollars) established for each market basket category (i.e., food group and subgroup) [10]. Categories include vegetables (dark-green, red and orange, beans, peas, lentils, starchy, and others), fruit (whole, 100% juice), grains (whole and refined), dairy (low to higher fat milk, yogurt and soy alternatives, cheese), protein foods (meats, poultry, eggs, seafood, nuts, seeds, and soy products) and miscellaneous (coffee, tea, table fats and oils, sauces, condiments, spices, etc.). Amounts of each food ingredient were standardized using the Food Patterns Equivalents Database (FPEDS) Portions and Weights table with the exception of brown rice and oatmeal [20]. In the FPED Portions and Weights table, brown rice and oatmeal are expressed only as cooked portions and weights, whereas the market basket quantities are expressed as dry weight. Therefore, the dry equivalents for the rice and oatmeal portions were standardized using FoodData Central [21]. Each ingredient amount was then converted to pounds and applied to its respective Market Basket cost to determine the cost per ingredient. The total cost of the one-day Thrifty Food Plan model was calculated from the summation of ingredient costs.

## 3. Results

### 3.1. CFQS Model Application to Food Items from Different Dietary Patterns

Approximately 20 CFs were selected to represent commonly consumed foods in the United States and applied across the four menu models described (Healthy US-Style, Healthy Vegetarian, Healthy Mediterranean-Style, and Thrifty Food Plan) (Table 1). These CFs were similar across all these dietary patterns since they all reflect general US-centric eating styles based on similar CF recommendations (e.g., aim for 45–65% kcal from carbohydrates and a similar number of daily servings of fruits, vegetables, and grains). Additionally, to reflect the diverse makeup of the US population, which is not well-captured in the three DGA-recommended Healthy Dietary Patterns, and to demonstrate the limitations of current US-centric DGA dietary guidance, we included four food tables based on culturally inclusive dietary patterns (African American, Latin American, Asian American, and Native American). These tables highlight CFs reflective of those cultures across the different carbohydrate-dominant food groups (fruits, vegetables, and whole grains) as well as for mixed dishes (Table 2, Table 3, Table 4 and Table 5).

### 3.2. CFQS Model Application to Existing DGA Healthy Dietary Patterns

For the Healthy US-Style Dietary Pattern (Table 6), the one-day sample menu was composed of three meals and two snacks and consisted of 2082 kcal. Macronutrient composition was 278 g carbohydrates (53% of kcal), 101 g protein (19% of kcal), and 68 g fat (29% of kcal). For CFQS nutrients, the sample menu provided 26 g fiber (93% of AI), 5 g added sugars (<10% of kcal), 3935 mg potassium (151% of the AI), and 1136 mg sodium (49% of RDA). The menu model was based on requirements for a woman, aged 20–50 years, consuming 2000 kcals/day.

For the Healthy Vegetarian Dietary Pattern (Table 7), the one-day sample menu was composed of three meals and two snacks and consisted of 2096 kcal. Macronutrient composition was 234 g carbohydrates (45% of kcal), 102 g protein (19% of kcal), and 92 g fat (40% of kcal). For CFQS nutrients, the sample menu provided 23 g fiber (81% of AI), 7 g added sugars (<10% of kcal), 3203 mg potassium (97% of the AI), and 2228 mg sodium (97% of RDA). The menu model was based on requirements for a woman, aged 20–50 years, consuming 2000 kcals/day.

For the Healthy Mediterranean-Style Dietary Pattern (Table 8), the one-day sample menu was composed of three meals and two snacks and consisted of 2094 kcal. Macronutrient composition was 251 g carbohydrates (48% of kcal), 125 g protein (24% of kcal), and 72 g fat (31% of kcal). For CFQS nutrients, the sample menu provided 31 g fiber (109% of AI), 0 g added sugars (<10% of kcal), 4787 mg potassium (184% of the AI), and 2025 mg sodium (88% of RDA). The menu model was based on requirements for a woman, aged 20–50 years, consuming 2000 kcals/day.

### 3.3. CFQS Model Application to the USDA Thrifty Food Plan

For the USDA Thrifty Food Plan (Table 9), the one-day sample menu was composed of three meals and two snacks and consisted of 2100 kcal. Macronutrient composition was 246 g carbohydrates (47% of kcal), 145 g protein (28% of kcal), and 65 g fat (28% of kcal). For CFQS nutrients, the sample menu provided 37 g fiber (130% of AI), 5 g added sugars (<10% of kcal), 4801 mg potassium (185% of the AI), and 1809 mg sodium (79% of RDA). The menu model was based on requirements for a woman, aged 20–50 years, consuming 2000 kcals/day. The menu model was based on average food costs for May 2022: Family cost/day = $7.63 per person. 1-person household (add 20%) = $9.15 per day.

## 4. Discussion

Currently, the carbohydrate intake recommendations in the DGA are primarily based on quantitative measures (i.e., RDAs, AMDRs, fiber content, and added sugars), with limited guidance provided on carbohydrate-containing food group qualities such as to select vegetables based on their color (e.g., dark-green, red, or orange) or starch-levels (i.e., starchy vegetables) and to select fruits and grains based on their “wholeness” (i.e., whole fruit or whole grains). Given these limitations regarding carbohydrate intake recommendations, along with the need to help policymakers, health professionals, and consumers to improve CF quality intake and overall diet quality in the US [2], new tools are necessary to assist in the identification and selection of higher from lower quality foods to help improve diet-related health outcomes. The CFQS-4 and CFQS-5 models were developed to provide a straightforward and easy-to-use tool that can help distinguish important aspects of carbohydrate quality, which are applicable to different dietary patterns and adaptable to new data sets.

When the CFQS models are applied to different foods, as either a CFQS-4 for non-grain CFs or CFQS-5 for grain foods, it can help distinguish which CFs should be more highly encouraged within the context of healthy dietary patterns to meet DGA recommendations regarding multiple nutrients of concern. In the present CF quality modeling exercises, various foods and sample menus were scored by the CFQS models to demonstrate their ease of use, alignment with current DGA recommendations, and applicability to other non-DGA dietary patterns that focus on factors such as economics and cultural/traditional influences in addition to nutrition.

### 4.1. Application of the CFQS Models to the 2020–2025 DGA Healthy Dietary Patterns

The aim of the Carbohydrate Food Quality Scoring System is to provide a tool that can guide policy, programs, and people towards improved food selection patterns. This scoring system has been specifically developed to align with four (for the CFQS-4) or five (for the CFQS-5) of the key healthy messages of the 2020–2025 DGA and could therefore serve as a basis for the development of much more useful and informative messaging than what is used currently. At present, DGA guidance on CFs is generally based on single variables such as “fiber”, “added sugars”, “starchy”, “whole”, or “dark-green; red and orange”. These recommendations do not provide very useful ways to compare carbohydrate quality, especially for starchy vegetables. In contrast, these new CFQS models have been specifically designed to align with multiple DGA recommendations that are associated with beneficial and proven health outcomes.

For the age–sex group (Females; 19–50 years; 2000 kcal/day) represented in this analysis, DGA guidance on CFs is rather consistent among the three recommended Healthy Dietary Patterns (e.g., 2.5 servings of vegetables, 2 to 2.5 servings of fruits, 6 to 6.5 servings of grains) [1]. The only difference between the CF recommendations of the different dietary patterns is that the Healthy Vegetarian Dietary Pattern includes 0.5 more servings of whole grains, and the Healthy Mediterranean-Style Dietary Pattern includes 0.5 more servings of fruits, than the other two patterns. Therefore, the selected CFs used in Table 1 can represent each of these dietary patterns. Despite these similarities, the foods included in the sample menus that were developed and analyzed for the modeling exercises varied considerably since they were able to be combined into many different recipes while still maintaining the overall nutritional integrity of the dietary pattern.

The application of the CFQS models to the 2020–2025 DGA Healthy US-Style, Vegetarian, and Mediterranean-Style Dietary Pattern menu models (Table 6, Table 7 and Table 8) demonstrate that both higher-scoring (CFQS ≥ 3) and lower-scoring (CFQS ≤ 2) CFs can fit into healthy dietary patterns that meet DGA recommendations for food and nutrient intake. On average, there are roughly five eating occasions per day in a US dietary pattern (three meals and two snacks), which means that there are ample opportunities to incorporate higher-quality CFs into different meals and snacks. Over the course of a day, our sample ‘healthy-style’ menu models (Table 6, Table 7, Table 8 and Table 9) indicate that a large variety of CFs from different food groups, encompassing the full range of CFQS scores, can be consumed to meet DGA nutrient intake recommendations. Many of the lower-scoring CFs may meaningfully contribute to one or more nutrients of concern of underconsumption to a dietary pattern and should therefore not automatically be considered discretionary. For example, while several enriched and fortified grains receive lower scores since a large portion of their nutrient contributions (e.g., iron, B-vitamins, and vitamins A, C, and D) may not currently be captured in the CFQS models, the DGA does recommend regular intakes of these foods (i.e., three servings/day) to help meet several nutrient needs for Americans [1,6]. Therefore, the public health messaging should not necessarily exclude all lower-scoring CFs from the diet since many of these, such as fortified and enriched grains, are vehicles for achieving nutrient adequacy for several micronutrients. Rather, the public health messaging might be more effective if it focused on making sure that consumers include a variety of higher-scoring CFs in their diets to help better align consumption patterns with multiple DGA recommendations specific to nutrients of public health concern.

### 4.2. CFQS Model Application to the USDA Thrifty Food Plan and Culturally Inclusive Dietary Patterns

Although the development of improved food quality metrics and models is a step in the right direction, the overall value of foods to health and well-being extends far beyond their nutrient and bioactive compound content. There are several economic and sociocul-tural factors, such as food cost and traditional elements, which also impact the food–health relationship and deserve further attention. These factors are not well represented in current DGA recommendations despite more than 11% of the US population living below the poverty line [22] and nearly 40% being classified as minorities [11]. One tool that may be helpful for health professionals and budget-conscious consumers is the USDA Thrifty Food Plan, which is the least expensive of the four USDA-designed food plans (other food plans are the Low-Cost, Moderate Cost, and Liberal Food plans), and contains a variety of low-price, nutrient-dense foods, and beverages that can help support healthy food selec-tion on a limited budget [10]. Unfortunately, eating to meet the DGAs based on the Thrifty Food Plan requires knowledge regarding menu planning and the nutrient value of inex-pensive food alternatives, as well as the ability to read nutrition fact labels and compare costs across different food brands and quantities. Some of the lower-cost foods may also require greater food preparation time than more costly convenience alternatives. Recent immigrants, persons with English as a second language, and/or those with limited in-comes, likely need more guidance, tools, and support with strengthening these skills than the general population.

Additionally, immigrants transitioning to a different cultural, social, economic, and geographical environment, while still identifying with and adhering to a traditional healthy dietary pattern, can also be difficult. Data indicate that the dietary transition that occurs post-immigration also often leads to a decreased consumption of cultur-al/traditional healthy staple CFs in place of lower-quality CFs, and these changes correlate with an increased risk for chronic disease [23]. The US is becoming increasingly diverse. According to the US 2020 Census, the Diversity Index for the US is 61.1%, which is rough-ly a 7-point increase in diversity occurring over a single decade [24]. In 2020, White Amer-icans comprised nearly 60% of the US population, while Hispanic/Latino populations comprised over 18% and African American populations comprised over 12% [24]. How-ever, in the coming decades, the White population is expected to decline, and multiple minority populations are expected to grow significantly [25]. This trend is already occur-ring in some US states and territories (e.g., California, District of Columbia, Hawaii, New Mexico, and Puerto Rico), where the largest population groups are not White [26], thus not likely consuming the types of dietary patterns recommended in the 2020–2025 DGA [27]. Hispanic Americans, African Americans, Asian Americans, and Native Americans have a wide array of traditional cuisines that include many different types of staple CFs (e.g., cassava, calabaza, taro, amaranth, millet, sorghum, plantains, mung beans, seaweed) that fall outside of the mainstream White American dietary pattern (e.g., wheat bread, oatmeal, pasta, potatoes, white rice). Dietary patterns, especially for recent immigrants, also gener-ally include more fruits and vegetables, an emphasis on legumes and pulses as protein sources, and soups and stews, in comparison to the typical US pattern [28]. While the 2020–2025 DGA does recognize the increasing diversity and food preferences of the US population and notes in Guideline 2 that “Nutrient-dense culturally relevant foods and beverages are part of all of the food groups” and that “people can customize the Dietary Guidelines recommendations to suit their personal preferences, cultural traditions, and budget considerations [1]”, it is increasingly clear that many Americans have difficulties in identifying, ranking, and/or selecting nutrient-dense foods, and could use evi-dence-based tools that help them in the process of identifying which of those nutri-ent-dense foods may best fit their preferences, traditions, and budget.

The 2020–2025 DGA provide lists of foods from different food groups that include a variety of culturally diverse foods as examples for customizing the DGA framework [1]. However, overall, culturally diverse dietary patterns have not been adequately included in the evidence analysis or creation, of the DGAs [29]. For example, a recent report on the re-search reviewed by the 2020 Dietary Guidelines Advisory Committee indicated that the committee relied almost exclusively on research conducted in White populations and that over 90% of the systematic reviews that they evaluated did not account for factors such as race, ethnicity, or socioeconomic status [29]. The 2020–2025 DGA did attempt to include a culturally diverse dietary pattern in their modeling; however, The Healthy Mediterrane-an-Style Dietary Pattern of the DGAs is essentially an adaptation of a European diet from the white southern countries of Greece, Italy, and Spain, not adequately representative of the twenty-one countries and three continents that are geographically part of the Mediter-ranean region [30]. Further inclusion in the dietary guidelines of non-White and non-European dietary patterns that include a wider range of more culturally diverse foods could go a long way towards addressing health inequities in the US, namely the dispro-portionate impact of chronic health conditions, insufficient food access, and socioeco-nomic conditions of underrepresented populations.

In the Thrifty Food Plan menu analysis (Table 9) and Culturally Inclusive Food Ta-bles (Table 2, Table 3, Table 4 and Table 5), the application of the CFQS models to different CFs makes it easy to see that there are several lower-cost, higher-quality CF options available for many different types of dietary patterns that can be prioritized to help individuals and their families sim-ultaneously to meet their economic and nutritional goals. Since CFs tend to be more af-fordable sources of nutrients than protein- or fat-rich foods, tools that can help improve the identification and selection of higher-quality options are critical during times of eco-nomic uncertainty, increasing inflation, and/or persistent food insecurity.

### 4.3. Limitations, Opportunities, and Next Steps

The present modeling exercises demonstrate the feasibility of using CFQS models to better understand the value and variation of CF quality among different food groups and types of healthy dietary patterns. However, even though the CFQS models are based on multiple DGA recommendations that are each linked to beneficial health outcomes, we do not currently have data as to how these composite scores will relate to health outcomes. An important next step in the development of CFQS-based tools would be to conduct research on the health impacts of consuming higher-scoring CFQS dietary patterns compared to lower-scoring patterns. Additionally, foods possess many “qualities” that can impact health, and these models currently only assess four or five of them. The CFQS-4 and CFQS-5 models put a premium on nutrients of public health concern, but at the same time, they end up omitting other key factors that are associated with health, such as the bioavailability of these nutrients, the content of other critical nutrients, and the composition and combinations of bioactive phytonutrients (e.g., anti-nutrients, antioxidants, prebiotics, probiotics). Further development of the CFQS models to assess various non-nutritive properties (e.g., bioactive properties) of food is warranted and deserves future attention once adequate research has been conducted and comprehensive databases have been developed to characterize the content of these compounds in different foods and to better understand their roles in health and well-being. Another limitation of the CFQS models is that the data sourced from current databases for inclusion in the models may not accurately capture the nutrient content of foods as they are commonly consumed. For example, the Food and Nutrient Database for Dietary Studies (FNDDS) database shows that many canned bean varieties are high in sodium; however, research shows that draining and rinsing canned beans before preparation may reduce the sodium content up to 40% or more [31]. Therefore, the act of draining and rinsing sodium-rich canned foods before consuming them could potentially increase their CFQS-4 score by an additional point, but this score change would not be captured by the current CFQS methodology in use.

A greater understanding of how non-Western staple CFs can impact nutrition and health within the context of culturally inclusive diets could also help improve dietary guidance for diverse populations. Current DGA-recommended Healthy Dietary Pattern models, which are based on only three different patterns, are insufficient for capturing the diversity of dietary patterns commonly consumed across the US and its territories. The DGA also focuses recommendations on food groups and subgroups and not on specific foods or beverages to avoid being prescriptive, leaving it up to the consumer to select their own healthy foods, beverages, and meals specific to their needs and preferences [1]. However, this leaves many culturally popular CFs (e.g., starchy vegetables, red/orange vegetables, legumes) classified in ways (e.g., by starch content or color) that make every food item within each food group or subgroup seem equivalent, when there is significant nutritional heterogeneity among foods in each food group and subgroup. This lack of nuance impairs consumers’ abilities to compare and select CFs based on their most important nutritional qualities. A more systematic method for identifying the quality of CFs, such as the CFQS models presented herein, could greatly support the overall goals of the DGA to improve diet quality for Americans.

## 5. Conclusions

The Carbohydrate Food Quality Scoring System is a multi-faceted CF quality assessment tool that can help reveal some of the important nuances associated with carbohydrate-rich foods since it does not depend only on a single nutrient (e.g., fiber) or a rather unpredictable physiological impact (e.g., glycemic index) to assess CF quality. Rather, the scoring system incorporates evidence-based cutoffs for multiple DGA nutrients of concern (added sugars, fiber, potassium, and sodium) that are associated with improved nutrition and health outcomes. The application of CFQS-4 and CFQS-5 metrics can therefore help inform policymakers, health professionals, consumers, and other food and nutrition stakeholders of the spectrum of CF quality available, both within and among food groups, with the overall goal of helping improve food selection for a variety of different dietary patterns, including DGA-promoted dietary patterns, the USDA Thrifty food plan, and more culturally inclusive dietary patterns.

A key output of these modeling exercises is the finding that both higher-scoring and lower-scoring CFs can be included in a variety of different healthy dietary patterns that meet DGA guidelines. While recommendations should focus on including a greater number of higher-scoring CFs in a dietary pattern, it should also be recognized that certain lower-scoring CFs have potential value for meeting nutrient demands, in addition to their roles as affordable staple foods in lower-cost and culturally inclusive dietary patterns. Overall, the CFQS models are easy-to-use CF quality assessment tools that further bring to life DGA guidance regarding CFs and could therefore help align public health policy, food industry actions, and consumer decisions to improve overall diet quality and nutrition-related health outcomes in the US.

## Figures and Tables

**Table 1:** CFQS Models Applied to Foods that Align with DGA—Healthy Dietary Patterns and the USDA Thrifty Food Plan.

USDA/DGA Carbohydrate Foods Examples	CFQS-4	CFQS-5
**Grains**		
Bread, sourdough		1
Crackers, whole wheat, low sodium		4
Granola		4
Noodles, whole wheat, cooked		4
Oatmeal, quick prepared, without salt		5
Pita bread, whole wheat		5
Popcorn, air popped, unsalted		5
Quinoa, cooked		5
Rice, brown, cooked, long grain		2
Spaghetti, whole wheat, cooked		3
Tortilla chips, white, corn		2
Tortilla, flour, 7″		1
**Vegetables**		
Arugula, fresh	4	
Bell pepper, red, fresh	3	
Carrots, fresh	4	
Cucumber, fresh, with skin	4	
Eggplant, fresh	3	
Green beans, boiled, drained	3	
Greens, spring mix	4	
Mushrooms, fresh	3	
Onion, red, fresh	3	
Onion, white, fresh	3	
Potato, boiled, peeled, small	3	
Potato, russet, fresh, with skin	3	
Potato, wedges, from frozen	3	
Spinach, fresh, leaf	4	
Sweet potato, baked, in skin, peeled	4	
Tomatoes, fresh, medium	4	
Zucchini, boiled, with skin	4	
**Fruits**		
Apple, fresh	4	
Blueberries, fresh	4	
Cantaloupe, fresh	3	
Orange, fresh, medium	4	
Peach, fresh, small	4	
Raisins	3	
Strawberries, fresh, sliced	4	
**Legumes**		
Black beans, canned, low sodium	4	
Cannellini beans, canned, no added fat	4	

**Table 2:** CFQS Models Applied to Latin American Culturally Inclusive Dietary Patterns.

Latin American—Carbohydrate Food Examples	CFQS-4	CFQS-5
**Grains**		
Tortilla, corn		4
Tortilla, whole wheat		4
Rice, yellow		2
Rice, white		1
Cornmeal, refined		2
**Vegetables**		
Beans, black or pinto (canned or dried)	4	
Beans, refried	3	
Chickpeas (canned, regular, or reduced sodium)	4	
Potato (boiled, peel eaten, made with oil)	4	
Tomatillos (fresh)	4	
Tomatoes (canned, reduced sodium, cooked)	4	
Kale/Dark Leafy Greens (cooked, no-added fat)	3	
**Fruits**		
Mango	4	
Plantains	3	
Cassava	3	
Guava	4	
**Mixed Dishes and Desserts**		
Tamale		2
Churro		1
Rice Pudding (arroz con leche)		2
Flan		2

**Table 3:** CFQS Models Applied to African American Culturally Inclusive Dietary Patterns.

African American-Carbohydrate Food Examples	CFQS-4	CFQS-5
**Grains**		
Biscuit, wheat		2
Millet (cooked with or without fat)		3
Grits (prepared with water or milk)		1
**Vegetables**		
Lima beans, Butter beans (from dried)	3	
Black-eyed peas, Split peas (from dried)	3	
Yam, Sweet Potato (baked, peel eaten, butter)	3	
Potatoes (roasted, peel eaten, no salt added)	4	
Collard Greens (fresh, cooked)	3	
Okra (fresh, cooked)	3	
Green beans (from frozen, cooked, no-fat)	3	
Beets (canned, reduced sodium, cooked)	4	
**Fruits**		
Melon (watermelon, cantaloupe, honeydew)	3	
Grapefruit	4	
Peach	4	
Papaya	4	
Grapes	3	
**Mixed Dishes and Desserts**		
Beans and Rice		3
Macaroni and cheese		1
Pie, sweet potato		2
Cobbler, peach		1

**Table 4:** CFQS Models Applied to Asian American Culturally Inclusive Dietary Patterns.

Asian American—Carbohydrate Food Examples	CFQS-4	CFQS-5
**Grains**		
Buckwheat noodles (buckwheat groats)		4
Noodles, soba, cooked (soup mostly noodles)		1
Chapati/Roti		5
Naan		3
Rice, basmati		1
**Vegetables**		
Mung beans (cooked)	3	
Mushrooms (cooked)	3	
Cabbage (cooked)	3	
Broccoli (cooked)	3	
Cellophane/mung bean noodles	2	
**Fruits**		
Mango	3	
Lychee	3	
Asian Pear	4	
Pineapple	4	
**Mixed Dishes and Desserts**		
Bibimbap		3
Congee, Asian, with meat & vegetable		2
Dumpling/Mandu, plain, steamed, bun		1
Mooncake, with lotus seed paste filling		1

**Table 5:** CFQS Models Applied to Native American Culturally Inclusive Dietary Patterns.

Native American—Carbohydrate Food Examples	CFQS-4	CFQS-5
**Grains**		
Wild Rice (cooked, no fat or salt added)		3
Oatmeal, rolled		5
Hominy (cooked)		2
Maize		3
**Vegetables**		
Corn (cooked)	4	
Turnip (cooked)	3	
Green Beans (cooked)	3	
Squash, yellow (cooked)	3	
Carrots, raw	4	
Beans, pink (cooked)	4	
Seaweed, raw	2	
**Fruits**		
Cranberries	4	
Persimmon	1	
Prickly Pear	4	
**Mixed Dishes and Desserts**		
Pancake, whole grain		2
Fry Bread		2
Cornbread		2

**Table 6:** Sample Daily Menu: Healthy US-Style.

Food Description	Amount	CFQS-4	CFQS-5
**Breakfast: Oatmeal with raisins; Scrambled eggs; Diced potato; Milk**			
Oatmeal, quickly prepared, without salt	4 oz		5
Raisins	1 oz	3	
Egg, scrambled	0.5 cup		
Canola oil	1 tbs		
Potato, russet, fresh, with skin, diced	0.5 cup	3	
Milk, nonfat, with vitamins A&D	1 cup		
Snack 1: Yogurt with Granola; Apple Slices			
Yogurt, Greek, plain, nonfat	0.5 cup		
Granola	1 oz		4
Apple, fresh, sliced	1 cup	4	
**Lunch: Black bean & rice bowl with tomatoes, cucumber, onion, tortilla chips; Orange**			
Rice, brown, cooked, long grain	1 cup		2
Black beans, canned, low sodium	0.5 cup	4	
Tomatoes, fresh, medium	0.5 each	4	
Cucumber, fresh, with skin, sliced	0.5 cup	4	
Tortilla chips, white, corn	1 oz		2
Orange, fresh, medium	2.5 oz	4	
Onion, white, fresh, chopped	0.25 cup	3	
**Dinner: Lemon chicken with spinach; Sweet potato; Milk**			
Chicken breast, roasted, skinless	3 oz		
Sweet potato, baked, in skin, peeled	0.5 cup	4	
Spinach, fresh, leaf	1 cup	4	
Canola oil	1 tbs		
Lemon juice	1 tbs		
Milk, nonfat, with vitamins A&D	1 cup		
**Snack 2: Cheese and crackers; Popcorn**			
Popcorn, air popped, unsalted	2 cups		5
Crackers, whole wheat, low sodium	7 each		4
Cheese, American, with vitamin D	0.5 oz		

**Table 7:** Sample Daily Menu: Healthy Vegetarian.

Food Description	Amount	CFQS-4	CFQS-5
**Breakfast: Breakfast Burrito (scrambled eggs, cheese, vegetables); Potatoes; Blueberries**			
Tortilla, flour, 7″	1 each		1
Eggs, scrambled	0.5 cup		
Cheese, American, with Vitamin D	0.75 oz		
Bell pepper, red, fresh, chopped	0.25 cup	3	
Onion, white, fresh, chopped	2 tbs	3	
Spinach, fresh, chopped	1 cup	4	
Potato, wedges, from frozen	1 oz	3	
Olive oil	0.5 tbs		
Blueberries, fresh	0.5 cup	4	
**Snack 1: Apple slices with peanut butter; Whole wheat crackers; Milk**			
Apple, fresh	0.5 each	4	
Peanut butter	1 tbs		
Crackers, whole wheat	10 each		4
Milk, nonfat, with vitamins A&D	1 cup		
**Lunch: Tempeh and quinoa salad with tortilla chips; Iced tea**			
Greens, spring mix	1 cup	4	
Tempeh	2 oz		
Quinoa, cooked	0.5 cup		5
Tomatoes, fresh, sliced	0.25 cup	4	
Cucumber, fresh, with skin, sliced	0.25 cup	4	
Cheese, cheddar, low-fat, shredded	1 oz		
Tortilla chips, white, corn	1 oz		2
Olive oil	0.5 tbs		
Lime juice, fresh	0.5 tbs		
Cumin seeds	0.5 tsp		
Paprika	0.5 tsp		
Black pepper, ground	1/8 tsp		
Iced tea, unsweetened	1 cup		
**Dinner: Tofu noodle stir fry; Milk**			
Tofu, firm	2 oz		
Noodles, whole wheat, cooked	0.75 cup		4
Carrots, fresh, sliced	0.33 cup	4	
Mushrooms, fresh, sliced	0.33 cup	3	
Onion, white, fresh, chopped	2 tbs	3	
Garlic cloves, fresh	1 each		
Sesame oil	1 tbs		
Ginger root, fresh	1 tbs		
Milk, nonfat, with vitamins A&D	5 oz		
**Snack 2: Frozen yogurt with strawberries and chocolate**			
Yogurt, Greek, plain, nonfat	0.75 cup		
Strawberries, fresh, sliced	0.5 cup	4	
Chocolate, dark	0.5 oz		

**Table 8:** Sample Daily Menu: Healthy Mediterranean-Style.

Food Description	Amount	CFQS-4	CFQS-5
**Breakfast: Hard boiled eggs with zucchini and feta cheese; Strawberries; Toast**			
Eggs, hard-boiled	2 each		
Zucchini, boiled, with skin, sliced	1 cup	4	
Cheese, feta, crumbled	1 oz		
Strawberries, fresh, sliced	1 cup	4	
Olive oil	0.5 tbs		
Bread, sourdough	2.5 oz		1
**Snack 1: Pita bread with hummus; Orange**			
Pita bread, whole wheat	1.5 oz		5
Hummus	2 tbs		
Orange, fresh, sections	0.75 cup	4	
**Lunch: Tuna niçoise salad; Potatoes; Milk**			
Potato, boiled, peeled, small	1 each	3	
Tuna, light, canned	2 oz		
Arugula, fresh, chopped	1 cup	4	
Cannellini beans, canned, no added fat	0.5 cup	4	
Green beans, boiled, drained	0.5 cup	3	
Olive oil	1 tbs		
Lemon juice, fresh	1 tsp		
Black pepper, ground	1/8 tsp		
Milk, 1%	1 cup		
**Dinner: Balsamic chicken; Spaghetti with roasted vegetables; Milk**			
Chicken breast, roasted, skinless	4 oz		
Spaghetti, whole wheat, cooked	1 cup		3
Spinach, fresh, chopped	1 cup	4	
Olive oil	1 tbs		
Balsamic vinegar	1 tbs		
Eggplant, fresh, cubes	0.5 cup	3	
Onion, red, fresh, sliced	0.25 cup	3	
Zucchini, fresh, with skin, chopped	0.5 cup	4	
Milk, nonfat, with vitamins A&D	1 cup		
**Snack 2: Mixed vegetables with mozzarella cheese**			
Tomatoes, cherry, red, fresh	9 each	4	
Cucumber, fresh, with skin, chopped	0.25 cup	4	
Cheese, mozzarella, part skim	1 oz		
Onion, red, fresh, sliced	0.25 cup	3	

**Table 9:** Sample Daily Menu: Thrifty Food Plan.

Food Description	Amount	CFQS-4	CFQS-5	Cost
**Breakfast: Hard boiled eggs with diced potato; Oatmeal**				
Egg, hard-boiled	2 each			$0.33
Potato, russet, fresh, with skin, medium	0.5 each	3		$0.37
Oatmeal, quick, prepared with water, without salt, unenriched	4 oz		5	$0.79
**Snack 1: Greek yogurt with almonds, fruit, and granola**				
Yogurt, Greek, plain, nonfat	0.5 cup			$0.16
Peach, fresh, small	1 each	4		$0.36
Cantaloupe, fresh, cubes	1 cup	3		$0.37
Almonds, whole	0.5 oz			$0.08
Granola	0.25 cup		4	$0.21
**Lunch: Chicken, black bean, and rice bowl; Milk**				
Chicken, ground, cooked	3 oz			$0.47
Cheese, cheddar, shredded	0.5 oz			$0.06
Rice, brown, cooked, medium grain	1 cup		2	$1.05
Black beans, canned, low sodium	0.25 cup	4		$0.09
Onion, white, fresh, chopped	0.25 cup	3		$0.12
Milk, nonfat, with vitamins A and D	6 oz			$0.24
**Dinner: Tilapia; Pasta with vegetables; Milk**				
Tilapia, baked	5 oz			$1.36
Pasta, spaghetti, cooked, enriched	1 cup		1	$0.49
Canola oil	1 tbs			$0.07
Carrots, boiled, drained, sliced	1 cup	4		$0.52
Spinach, boiled, from frozen	1 cup	4		$0.86
Lemon juice, fresh	2 tbs			$0.07
Salt, table	0.8 tsp			$0.01
Milk, nonfat, with vitamins A&D	1 cup			$0.32
Tomatoes, cherry, red, fresh	4 each	4		$0.23
**Snack 2: Cheese; Popcorn**				
Cheese, American, with vitamin D	0.5 oz			$0.07
Popcorn, air popped, unsalted	2 cups		5	$0.09

## Data Availability

Not applicable.
